# Physical Properties of Polyamide-12 versus PMMA Denture Base Material

**DOI:** 10.1155/2014/150298

**Published:** 2014-03-09

**Authors:** Mieszko Wieckiewicz, Volker Opitz, Gert Richter, Klaus W. Boening

**Affiliations:** ^1^Division of Dental Materials, Faculty of Dentistry, Wroclaw Medical University, 26 Krakowska street, 50-425 Wroclaw, Poland; ^2^Department of Prosthetic Dentistry, Faculty of Medicine, Dresden University of Technology, Fetscherstraße 74, 01307 Dresden, Germany

## Abstract

*Objectives.* Polyamide-12 (PA) is a flexible material suited for denture bases and clasping. This study investigated its potential aging effects with a focus on surface roughness, color stability, and elasticity. *Methods.* PA specimens (Valplast) of 40 × 10 × 2 mm and equally measuring PMMA specimens (Palapress) as control were fabricated. Color changes after storage in air, water, coffee, and red wine (*n* = 10) were measured using the CIE L*a*b* color specification. Elasticity after thermocycling (1000, 3000, and 7000 cycles,  *n* = 15) was measured by three-point bending testing. Mean surface roughness (Ra) was determined after storage in the liquids mentioned above and thermocycling (*n* = 10). *Results.* Tukey's HSD test (*P* < 0.05) revealed statistically significant color changes of PA in red wine (Δ*E* = 4.27 after 12 days, Δ*E* = 6.90 after 12 days) and coffee (Δ*E* = 3.93 after 36 days) but no color changes in PMMA. Elastic modulus of PA was 845 MPa and not affected by thermocycling (Tukey's HSD test,  *P* > 0.81). Dry specimens showed significantly decreased elasticity (*P* < 0.001). Mean surface roughness (PA 0.20 **μ**m, PMMA 0.28 **μ**m) did not change significantly after thermocycling or storage (Mann-Whitney *U*-test,  0.16 < *P* < 0.65). *Significance*. PA exhibited a higher susceptibility to discoloration than PMMA. Neither surface roughness nor elasticity of PA was altered by artificial aging.

## 1. Introduction

Despite the progress in dental implant treatment, there is still need for conventional removable partial dentures. Edentulism in the developed countries is in decline, but the number of patients suffering from partial tooth loss continues to rise [1–4]. On the other hand, in countries ranked lower in economic development and welfare index the rates of edentulism remain high [5–9]. Thus it has to be expected that the need for cost effective removable partial dentures will remain substantial.

**Figure 1:**
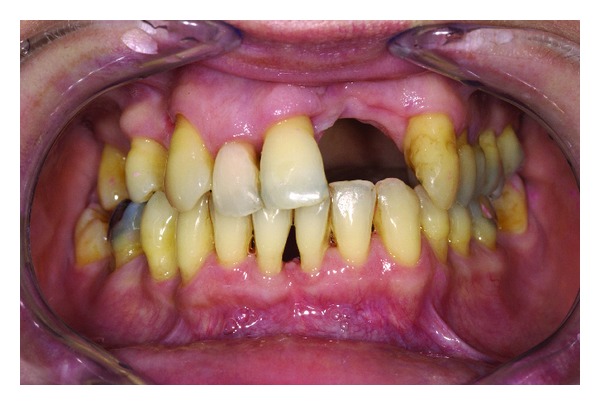
Upper jaw with missing central and lateral incisors in the second quadrant and large defect of the alveolar ridge.

**Figure 2:**
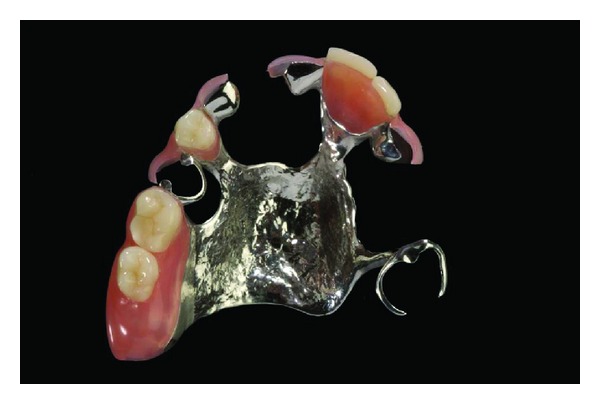
Removable partial denture with denture saddles and retentive clasping arms made from polyamide-12. The conventional CoCrMo framework ensures rigidity of the denture despite the flexibility of the denture resin material.

**Figure 3:**
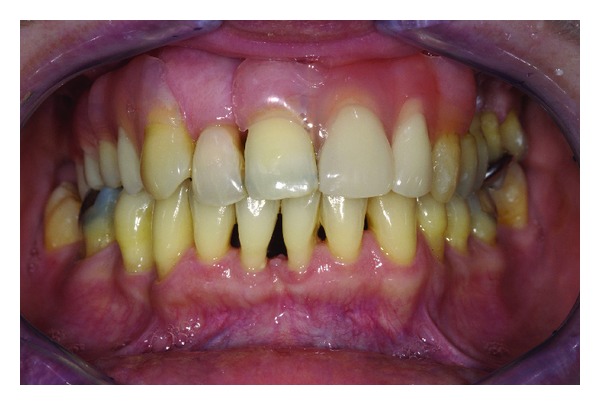
Removable partial denture in situ. The polyamide-12 retentive clasping arms are almost invisible.

Polymethyl methacrylate (PMMA) is commonly used to fabricate removable dentures. PMMA shows sufficient material properties and ease of application [10]. However, an increasing rate of intolerance to monomers present in acrylic materials among patients and medical staff has been reported [11, 12]. Furthermore, the aesthetic appearance of removable partial dentures with PMMA bases may be compromised by the visibility of metal clasps. A feasible alternative to PMMA-based removable partial dentures may be the use of polyamide-12 [13]. The flexibility of polyamide-12 allows retentive elements that match the color of the gums or teeth. Partial dentures might be pressed in one piece including clasps, minor and major connectors, and denture bases [14]. However, these nonrigid denture designs are discussed controversially since flexible bases may cause higher displacement of soft tissue and their influence on ridge resorption is not yet fully understood [15]. A solution could be the integration of a metal framework providing rigid major connectors and occlusal rests (Figures 1, 2, and 3).

Shortcomings of polyamide-12 discussed comprise a more complex process of polishing and inferior color stability [16, 17]. Particularly early nylon dentures exhibited high water sorption leading to rapid discoloration, loss of surface finish, and softening [18]. However, the conditions in which these dentures were processed were far from ideal [18]. The objective of this study was to investigate a modern polyamide-12 processed using specialized laboratory equipment for its potential discoloration, change in elasticity, and surface roughness in different storage media and after thermocycling. PMMA served as a control. The following null hypotheses were stated.

1. There is no difference in susceptibility to discoloration between polyamide-12 and PMMA.
2. Elasticity of polyamide-12 does not change with artificial aging.
3. There is no difference in surface roughness between polyamide-12 and PMMA.
4. Surface roughness in polyamide-12 and PMMA does not change with artificial aging.

## 2. Materials and Methods

Polyamide-12 specimens (Valplast, Valplast International Corp., Long Beach, NY, USA, LOT 111224) were fabricated, each measuring 40 × 10 × 2 mm. Wax patterns were embedded in formaldehyde-free stone (Weiton-Biogips Typ 4, Weithas Corp., Lütjenburg, Germany) and polyamide-12 was injected at a temperature of 290°C and a pressure of 6.5 bar according to the manufacturer's instructions using the Valplast injection system (Valplast International Corp., Long Beach, NY, USA). The polyamide specimens were polished with Tripoli-Paste and Val-Mirror-Shine polishing paste (Weithas Corp., Lütjenburg, Germany). PMMA specimens (Palapress, Heraeus Kulzer GmbH, Hanau, Germany, LOT 011093) were fabricated, each measuring 40 × 10 × 2 mm. Powder and liquid were mixed according to the manufacturer's instructions and cast into molds made from plaster (Modelit, Siladent Company, Goslar, Germany). Polymerisation was accomplished at 55°C and a pressure of 2.5 bar for 30 minutes in a pressure cooker (Polyclav, Dentaurum Corp., Ispringen, Germany). The specimens were smoothed with sandpaper (grit size 120) and prepolished with pumice slurry (Steribim, Bego, Bremen, Germany). Final polishing was done with cotton polishers (Polirapid, Singen, Germany) and Universal Polishing Paste (Ivoclar Vivadent, Schaan, Liechtenstein). All statistical analyses were conducted with STATISTICA ver. 10 software (StatSoft Inc., Tulsa, OK, USA).

### 2.1. Discoloration after Storage in Different Media

Discoloration of polyamide-12 and PMMA specimens was assessed using the Lab color space (CIE L*a*b*). The parameter L* represents the lightness of the color from 0 (black) to 100 (white), a* the color position between green (negative values) and magenta (positive values), and b* the color position between blue (negative values) and yellow (positive values). The two latter parameters range from −150 to +100 and from −100 to +150, respectively, regardless of the fact that some of the values do not represent an actual (visible) color.

Ten polyamide-12 specimens each were stored in air, distilled water, instant coffee (Nescafé Gold, Nestlé Frankfurt/Main, Germany), and red wine (König Arthur, Andreas Oster AG, Cochem, Germany) at a temperature of 37°C. An equal number of PMMA specimens served as control. Color measurements were conducted prior to storage and then after 24 hours, 12 days, and 36 days of storage. Due to potential fungal growth especially on coffee, all liquids were changed every three days. Glass grids ensured surface exposure to the surrounding media.

Discoloration tests were carried out using the Gretag SPM 100 spectrophotometer (Gretag Color Control Systems, Regensdorf, Switzerland) and the light box Highlight 2000 platform (OLBRICH know how, Hemer, Germany) to ensure standardized light conditions. Prior to the measurements, all specimens were thoroughly rinsed with distilled water and their surfaces were carefully dried with soft absorbent paper tissues. In each specimen measurements were obtained in four separate surface spots. Because of the textured colored polyamide-12 specimens and potential small color variation in the PMMA specimens, a template was made to ensure exact repositioning of all specimens in the measuring device and thus identical spots for repeated measurements.

Color differences were calculated according to the formula Δ*E* = [(ΔL*)^2^ + (Δa*)^2^ + (Δb*)^2^]^1/2^ [19, 20]. From the measurements on the four separate spots in each specimen one separate average value for  L*,  a*, and b* were calculated. ΔL*, Δa*, and Δb* were calculated from the initial measurements and the measurements after 24 hours, 12 days, and 36 days, respectively. The results were statistically analysed using the 3-way repeated measures analysis of variance (ANOVA) model with post hoc analysis using Tukey's HSD test (*P* = 0.05).

### 2.2. Elasticity of Polyamide-12 after Artificial Aging

**Figure 4:**
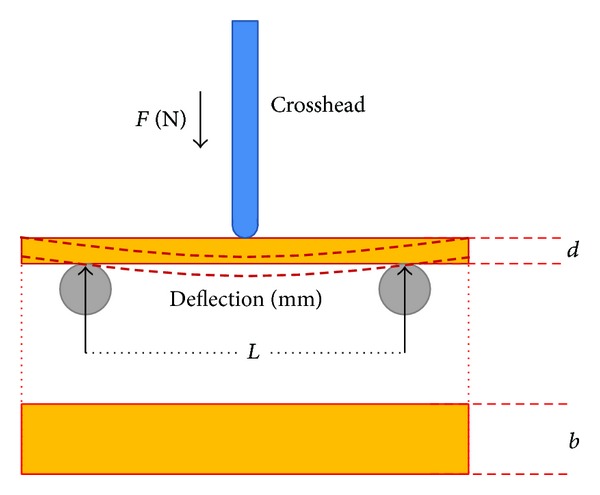
Three-point bending test to determine the modulus of elasticity in polyamide-12 specimens. *L*: support span (30 mm), *b*: specimen width (10 mm), *d*: specimen thickness (2 mm), and crosshead speed 1.0 mm/min (corresponding equation in the text).

The modulus of elasticity [21] was measured in polyamide-12 specimens using the three-point bending test (Figure 4) and calculating the modulus of elasticity according to the formula *E* = *L* ^3^ *m*/4*bd* ^3^ with

 
*E* being modulus of elasticity (N/mm^2^);
 
*L* being support span (mm);
 
*m* being gradient of the initial straight-line portion of the load deflection curve (N/mm);
 
*b* being width of tested specimen (mm);
 
*d* being thickness of tested specimen (mm).

A total of 15 polyamide-12 specimens were tested. All specimens were tested dry after they arrived from the dental laboratory and then after 1000, 3000, and 7000 cycles of thermocycling. Thermocycling was carried out in tab water at 5°C ±  2°C and 55°C ±  2°C, using the Festo FPC 101 Step Controller (Festo AG and Co. KG, Esslingen, Germany). Each cycle consisted of 27 seconds of submersion and a 15-second period in which the specimens were moved between the two containers. After thermocycling the specimens were stored in distilled water until testing. Three-point bending tests were carried out using the TIRAtest 2720 testing machine (TIRA GmbH, Schalkau, Germany). The data were analysed using 1-way repeated measures analysis of variance (ANOVA) model post hoc analysis using Tukey's HSD test (*P* = 0.05).

### 2.3. Surface Roughness after Storage in Different Media and Artificial Aging

Surface roughness was measured with a Hommel Tester T6000 profilometer (Hommelwerke GmbH, Villingen-Schwenningen, Germany) using a circular diamond stylus (*r* = 5 *μ*m, angle 90°). The stylus moved at 0.5 mm/s with a scan length range of 4 mm and a sampling force of 0.7 mN.

Mean surface roughness (Ra) was assessed in 10 polyamide-12 and 10 PMMA specimens shortly after delivery from the dental laboratory, that is, four to five days after polishing. Furthermore surface roughness was assessed in all specimens from the discoloration tests in water, coffee, and red wine after 36 days of storage and additionally in 10 polyamide-12 specimens and 10 PMMA specimens after 5000 times of thermocycling as described above. Prior to the measurements all specimens were thoroughly rinsed with distilled water and their surfaces were carefully dried with soft absorbent paper tissues. In each specimen, four different scans were carried out, two laterally and two vertically. From the four scans an average value was calculated for each individual specimen. Within the specimen groups polyamide-12 and PMMA mean surface roughness Ra was analysed using the Kruskal-Wallis test of variance. To compare Ra between polyamide-12 and PMMA (comparing specimens stored in the same conditions), Mann-Whitney *U* test was used (*P* = 0.05).

## 3. Results

### 3.1. Discoloration in Different Storage Media

The results are shown in Table 1. Testing the assumptions for the use of a repeated measures ANOVA for statistical analysis of discoloration showed deviations in the equality of variance and distribution for the studied groups. However, an ANOVA is fairly robust to the violations of both assumptions, especially when sample sizes in each group are equal [22] and sphericity assumption was verified (*W* = 0.92; *χ* ^2^(df = 2) = 5.65, *P* = 0.06).

The three-way analysis of variance for repeated measures with material (polyamide-12, PMMA), time (1, 12, and 36 days) media (air, water, coffee, and red wine) being the independent variables and Δ*E* being the dependent variable revealed significant differences [storage time ∗ material ∗ conditions *F*_(6,144)_ = 29.05, *P* < 0.0001]. So a post hoc analysis using Tukey's HSD test was carried out to identify significant differences.

In polyamide-12 specimens submerged in red wine, Δ*E* after 12 days and 36 days was statistically significantly higher compared to Δ*E* obtained after 24 hours (*P* < 0.05). In polyamide-12 specimens submerged in coffee, Δ*E* after 36 days was statistically significantly higher compared to Δ*E* obtained after 24 hours and after 12 days (*P* < 0.05). The differences between Δ*E* of the polyamide-12 specimens stored in coffee for 24 hours and 12 days were not statistically significant (*P* > 0.05) as well as after storage in air or water. Δ*E* for PMMA specimens was not statistically different regardless of the storage media or the submersion time (*P* > 0.05). Thus the null hypothesis of similar susceptibility to discoloration of polyamide-12 and PMMA had to be rejected.

### 3.2. Elasticity of Polyamide-12 after Artificial Aging

The values for modulus of elasticity obtained in this study are presented in Table 2. A one-way repeated measures ANOVA with modulus of elasticity being the dependent variable was used to search for any significant differences within the four groups *after delivery, after 1000, 3000, and 7000 thermocycles *and revealed significant differences [*F*(3.56) = 58,11; *P* < 0.0001]. A post hoc analysis using Tukey's HSD test was carried out to identify significant differences.

Elastic moduli after 1000, 3000, and 7000 thermocycles did not show any statistically significant differences (*P* > 0.81). Thus the null hypothesis of no changes of elasticity with artificial aging had to be accepted. However, Tukey's HSD test showed a statistically significant higher elastic modulus of dry specimens after delivery from the dental laboratory compared to all thermocycled specimens (*P* < 0.001).

### 3.3. Surface Roughness

Surface roughness values of polyamide-12 and PMMA are shown in Table 3. Polyamide-12 surface roughness (Ra) values were tested for normal distribution using the Shapiro-Wilk test. In the three groups *after delivery* (*W* = 0.79; *P* = 0.01), *after water storage* (*W* = 0.83; *P* = 0.04), and *after red wine storage* (*W* = 0.73; *P* = 0.01) the results were not distributed normally. Hence Ra values within the five polyamide-12 groups were tested for significant differences using the Kruskal-Wallis test which revealed no differences between the groups [*H*(4) = 2.26; *P* = 0.69].

PMMA surface roughness (Ra) values were also tested for normal distribution using the Shapiro-Wilk test. In the group *after water storage* (*W* = 0.73; *P* = 0.002) the results were not distributed normally. Thus significant differences in Ra values within the five PMMA groups were tested using the Kruskal-Wallis test which again revealed no differences between the groups [*H*(4) = 3.20; *P* = 0.52].

The Mann-Whitney *U* test was used to compare Ra values for the polyamide-12 groups and PMMA groups stored under the same conditions. Statistically significant differences could not be found between any of the groups (0.16 < *P* < 0.65). The two null hypotheses assuming similar surface roughness between polyamide-12 and PMMA and assuming no changes in Ra with artificial aging had to be accepted.

## 4. Discussion

### 4.1. Discoloration in Different Storage Media

If a denture design with retentive clasp arms from polyamide-12 is chosen primarily for aesthetic reasons, anterior resin material may be substantially more visible than in partial dentures with conventional bases and metal clasps. Thus resin discoloration over time may lead to unfavourable aesthetics especially in these prostheses. In this study red wine and coffee were selected because of their popularity and their staining potential [23, 24]. Coffee demonstrated to have a higher staining potential than tea [16, 24].

The CIE L*a*b* color specification is a popular method of measuring colors and is the basis of most color management systems and software [19, 20, 23]. It enables to compare color value changes in each of the specimen groups regardless of the external conditions occurring during assessment. The ability of the human eye to appreciate color differences may change from individual to individual. To evaluate the impact of color changes to aesthetics in dental materials, Vichi et al. [20] recommend three different intervals for distinguishing color differences Δ*E*. Values of Δ*E* < 1 were regarded as not appreciable by the human eye. Values 1 < Δ*E* < 3.3 were considered appreciable by skilled operators but considered clinically acceptable, whilst values of Δ*E* > 3.3 were considered appreciable also by nonskilled persons and for that reason clinically not acceptable. Sepulveda-Navarro et al. [23] used NBS (National Bureau of Standards) units to relate Δ*E* values to a clinical environment through the equation NBS units = Δ*E* × 0.92. Critical remarks of NBS units are 0.0–0.5 (trace), 0.5–1.5 (slight), 1.5–3.0 (noticeable), 6.0–12.0 (much) and ±12.0 (very much) [23].

Accepting a NBS unit of 3.0 (~Δ*E* of 3.3)as a threshold for clinically acceptable color change [25] it can be considered that all PMMA specimens were well in that range in this study. Polyamide-12 showed discoloration exceeding a Δ*E* of 3.3 after 12 days of storage in red wine and 36 days of storage in coffee. After 36 days in red wine discoloration exceeded the second threshold of Δ*E* = 6.52. Thus the most severe staining of the polyamide-12 specimens was apparent with red wine. The staining effect of coffee was significantly lower. This is in agreement with other studies in which the highest color difference for all restorative materials was observed in the red wine groups followed by tea or coffee [23, 24, 26, 27].

The stainability of polymers by coffee has been attributed to the presence of yellow colorants with different polarities and probably might be both adsorbed and absorbed due to compatibility of the polymer phase with the colorants. Particularly tannic acid has been proved responsible for the staining capability of coffee [28].

In red wine the alcohol content as well as a low pH seems significant in affecting the color stability in both acrylic and nylon denture base resins [29]. Studies have reported that the acidic pH may affect the material structure and that alcohol facilitates staining by softening the resin matrix [27, 28]. Since the pH of red wine in this study was 2.9 (Shindengen pH Boy-P2, Electric MFG., Tokyo, Japan) and the alcohol content 11% by volume, roughened denture base resin surfaces might have been expected, thereby resulting in increased staining. However, surface roughness was independent of storage media and storage time in this study.

Based on the literature and on the data in this study, 36 days of red wine storage might be considered a worst-case scenario for denture base materials (equalling 14 years with an assumed daily red wine exposure of 10 minutes). However, polyamide-12 seems to be more susceptible to discoloration by staining liquids compared to PMMA. On the other hand, it has been proven that adequate oral hygiene and professional care can substantially reduce the problem of staining [26, 28].

### 4.2. Elasticity of Polyamide-12 after Artificial Aging

Retentive arms with a constant flexibility over time are essential for a satisfactory function of polyamide-12 removable partial denture with nonmetal clasping.

In this study after artificial aging comprising 1000, 3000, and 7000 thermocycles the modulus of elasticity showed only miniscule differences. Mean values were between 828 and 848 MPa and statistically not significant. These data correspond to the literature where an elastic modulus of 826 MPa was reported for polyamide-12 from three-point bending tests after storage in water but without artificial aging [30].

In this study measurements shortly after delivery from the dental laboratory and prior to thermocycling revealed a significant higher elastic modulus of 1075 MPa. This might lead to the assumption of a softening effect of the thermocycling procedure on polyamide-12. However, these initial measurements were done on dry specimens, while measurements after 1000, 3000, and 7000 thermocycles were done on specimens stored wet throughout the experiments. Furthermore, pretests within this study showed a modulus of elasticity of 1113 MPa after 5000 thermocycles plus a dry storage of two days. Hence it might be speculated that the penetration of water molecules into the structure has a lowering effect on the modulus of elasticity in polyamide-12. The effects of water absorption have been discussed in the literature [21, 30–32]. Although further research is needed to evaluate the specific role of water absorption, the results underline the necessity of wet conditions when investigating mechanical properties of polyamide-12 with respect to use in the oral cavity.

The elastic modulus of PMMA was not investigated in this study because there is no clinical indication for PMMA as a material for retentive clasps in removable partial dentures.

### 4.3. Surface Roughness

Rough surfaces may cause discoloration of denture base material and may also contribute to biofilm formation because bacteria and fungus have more of a propensity to adhere to rough surfaces [33, 34]. Previous studies have shown that Ra = 0.2 might be accepted as a threshold level for surface smoothness of denture base materials for no further reduction in plaque accumulation is to be expected with more extensive polishing [35].

Technologically polishing is easier in PMMA rather than in polyamide-12. Normally PMMA surfaces are initially less rough than polyamide-12 due to its injection under pressure and high temperatures [17].

However, in this study no significant differences in surface roughness were found between PMMA and polyamide-12. Mean values of Ra for polyamide-12 ranged between 0.20 and 0.33 *μ*m while mean values of Ra for PMMA ranged between 0.20 and 0.28 *μ*m. Although the majority of the Ra values measured in this study were somehow close to the accepted Ra = 0.2 *μ*m mentioned above, Ra values of the PMMA specimens in this study were considerably higher compared to literature data [17] where mean values for Ra below 0.15 *μ*m (polyamide-12) and below 0.05 *μ*m (PMMA) were reported.

This may be attributed to the fact that the PMMA specimens in this study were manufactured by the authors who are experienced in the dental laboratory but not professional dental technicians while the polyamide-12 specimens were made in a commercial laboratory using specialized equipment. Furthermore the study cited [17] used a lathe for polishing while in this study common tools from the dental laboratory were used where speed and pressure of the rotating polishers are difficult to standardize [36]. This also may explain the high variability of Ra values measured.

Surprisingly artificial aging and storage in red wine and coffee did not significantly affect the surface roughness, neither in the PMMA nor in the polyamide-12 specimens. Theoretically a roughened surface might have been expected as a result of the exposure to attacking acids from coffee and red wine, a softening effect of ethyl alcohol (11% by volume in the red wine) [16, 23], or the temperature changes during thermocycling.

## 5. Conclusions

Within the limits of this study the results justify the use of polyamide-12 for denture saddles and retentive elements and thus allow the fabrication of cost effective but still aesthetic pleasing removable partial dentures where a conventional design with metal clasping would lead to poor aesthetics. However, polyamide-12 is less resistant to staining than PMMA. Hence clinicians should advise patients that drinking red wine could intensify surface staining on polyamide-12 prosthesis. Dry polyamide-12 is significantly less flexible. Thus polyamide-12 prostheses should be stored in water prior to insertion and patients should be advised accordingly.

## Figures and Tables

**Table 1:** Color changes Δ*E* in polyamide-12 and PMMA after storage in air, water, coffee, and red wine (*n* = 10).

Material	Storage medium	Storage time (days)	Mean ΔE	SE	Min	Max
Polyamide-12	Air	1	2.26	0.13	1.80	3.02
		12	2.01	0.06	1.67	2.26
		36	2.29	0.09	1.89	2.89
	Water	1	2.90	0.20	2.15	3.92
		12	2.23	0.10	1.84	2.84
		36	2.18	0.08	1.79	2.62
	Coffee	1	1.59^#^	0.08	1.20	1.98
		12	2.04^##^	0.19	1.35	3.51
		36	**3.93** ^ #,##^	0.29	2.35	5.30
	Red wine	1	2.40^∗,∗∗^	0.23	1.39	3.54
		12	4.27*	0.11	3.66	4.76
		36	6.90**	0.26	5.36	8.49

PMMA	Air	1	2.44	0.12	1.93	3.14
		12	2.06	0.05	1.84	2.35
		36	1.94	0.06	1.83	2.48
	Water	1	2.79	0.15	2.1	3.47
		12	2.67	0.15	2.05	3.56
		36	2.22	0.09	1.99	2.79
	Coffee	1	2.05	0.12	1.62	2.74
		12	2.02	0.18	1.52	3.46
		36	1.60	0.12	1.30	2.59
	Red wine	1	2.87	0.22	2.09	4.28
		12	2.76	0.18	2.22	3.82
		36	2.48	0.33	1.77	5.17

Appreciable color changes (Δ*E* > 3.3) in bold letters. Significant differences are indicated by ^#,##^(coffee), ^∗,∗∗^(red wine).

**Table 2:** Modulus of elasticity (MPa) of polyamide-12 specimens (*n* = 15). Modulus of elasticity of dry specimens after delivery from the dental laboratory differed significantly from values after 1000, 3000, and 7000 thermocycles and wet storage.

MPa	After delivery (dry storage)	After 1000 thermocycles (wet storage)	After 3000 thermocycles (wet storage)	After 7000 thermocycles (wet storage)
Mean	1084	828	848	845
SE	21	13	16	12
Min	870	708	742	780
Max	1178	885	981	960

**Table 3:** Mean surface roughness Ra (*µ*m) of polyamide-12 and PMMA after delivery from the dental laboratory, after storage in water, coffee, red wine (36 days), and thermocycling (*n* = 10). Significant differences were found neither within the polyamide-12 and PMMA groups nor between polyamide-12 and PMMA.

Procedure	Material	Mean Ra (µm)	SE	Min	Max
After delivery	Polyamide-12	0.28	0.05	0.07	0.42
	PMMA	0.20	0.02	0.13	0.33

Water	Polyamide-12	0.21	0.05	0.07	0.49
	PMMA	0.24	0.03	0.15	0.48

Coffee	Polyamide-12	0.33	0.07	0.09	0.76
	PMMA	0.26	0.03	0.14	0.44

Red wine	Polyamide-12	0.20	0.05	0.09	0.52
	PMMA	0.28	0.04	0.12	0.54

5000 thermocycles	Polyamide-12	0.29	0.07	0.07	0.66
	PMMA	0.24	0.02	0.16	0.33

## References

[1] Ettinger RL, Beck JD, Jakobsen J (1984). Removable prosthodontic treatment needs: a survey. *The Journal of Prosthetic Dentistry*.

[2] Vargas CM, Casper JS, Altema-Johnson D, Kolasny CR (2012). Oral health trends in Maryland. *Journal of Public Health Dentistry*.

[3] Lundegren N, Axtelius B, Akerman S (2011). Self perceived oral health, oral treatment need and the use of oral health care of the adult population in Skåne, Sweden. *Swedish Dental Journal*.

[4] Jokstad A (2002). Oral prosthetics from a Nordic perspective. *The International Journal of Prosthodontics*.

[5] Listl S, Moran V, Maurer J, Faggion CM (2012). Dental service utilization by Europeans aged 50 plus. *Community Dentistry and Oral Epidemiology*.

[6] Reddy NS, Reddy NA, Narendra R, Reddy SD (2012). Epidemiological survey on edentulousness. *The Journal of Contemporary Dental Practice*.

[7] Ariga P, Bridgitte A, Rangarajan V, Philip JM (2012). Edentulousness, denture wear and denture needs of the elderly in rural South India. *Iranian Journal of Public Health*.

[8] Sveikata K, Balciuniene I, Tutkuviene J (2012). Needs for prosthetic treatment in Vilnius population at the age over 45 years old. *Stomatologija*.

[9] Gaio EJ, Haas AN, Carrard VC, Oppermann RV, Albandar J, Susin C (2012). Oral health status in elders from South Brazil: a population-based study. *Gerodontology*.

[10] Rickman LJ, Padipatvuthikul P, Satterthwaite JD (2012). Contemporary denture base resins: part 1. *Dental Update*.

[11] Seppäläinen AM, Rajaniemi R (1984). Local neurotoxicity of methyl methacrylate among dental technicians. *American Journal of Industrial Medicine*.

[12] Gautam R, Singh RD, Sharma VP, Siddhartha R, Chand P, Kumar R (2012). Biocompatibility of polymethylmethacrylate resins used in dentistry. *Journal of Biomedical Materials Research B*.

[13] Singh JP, Dhiman RK, Bedi RP, Girish SH (2011). Flexible denture base material: a viable alternative to conventional acrylic denture base material. *Contemporary Clinical Dentistry*.

[14] Kaplan P (2008). Flexible removable partial dentures: design and clasp concepts. *Dentistry Today*.

[15] Zhou Z, Hu Y-D, Sui Q-S, Yan N-J, Ye R (2011). Application of valplast dentures in the temporary restoration of single missing anterior tooth. *Acta Academiae Medicinae Sinicae*.

[16] Buyukyilmaz S, Ruyter IE (1994). Color stability of denture base polymers. *The International Journal of Prosthodontics*.

[17] Abuzar MA, Bellur S, Duong N (2010). Evaluating surface roughness of a polyamide denture base material in comparison with poly (methyl methacrylate). *Journal of Oral Science*.

[18] Rickman LJ, Padipatvuthikul P, Satterthwaite JD (2012). Contemporary denture base resins: part 2. *Dental Update*.

[19] Bureau central de la CIE (1978). *Recommendations on Uniform Color Spaces, Color-difference Equations, Psychometric Color Terms*.

[20] Vichi A, Ferrari M, Davidson CL (2004). Color and opacity variations in three different resin-based composite products after water aging. *Dental Materials*.

[21] Ucar Y, Akova T, Aysan I (2012). Mechanical properties of polyamide versus different PMMA denture base materials. *Journal of Prosthodontics*.

[22] Field A (2005). Exploring data. *Discovering Statistics Using SPSS*.

[23] Sepulveda-Navarro WF, Arana-Correa BE, Borges CP, Jorge JH, Urban VM, Campanha NH (2011). Color stability of resins and nylon as denture base material in beverages. *Journal of Prosthodontics*.

[24] Guler AU, Yilmaz F, Kulunk T, Guler E, Kurt S (2005). Effects of different drinks on stainability of resin composite provisional restorative materials. *The Journal of Prosthetic Dentistry*.

[25] Um CM, Ruyter IE (1991). Staining of resin-based veneering materials with coffee and tea. *Quintessence International*.

[26] Omata Y, Uno S, Nakaoki Y (2006). Staining of hybrid composites with coffee, oolong tea, or red wine. *Dental Materials Journal*.

[27] Rutkunas V, Sabaliauskas V, Mizutani H (2010). Effects of different food colorants and polishing techniques on color stability of provisional prosthetic materials. *Dental Materials Journal*.

[28] Bagheri R, Burrow MF, Tyas M (2005). Influence of food-simulating solutions and surface finish on susceptibility to staining of aesthetic restorative materials. *Journal of Dentistry*.

[29] Patel SB, Gordan VV, Barrett AA, Shen C (2004). The effect of surface finishing and storage solutions on the color stability of resin-based composites. *The Journal of the American Dental Association*.

[30] Hamanaka I, Takahashi Y, Shimizu H (2011). Mechanical properties of injection-molded thermoplastic denture base resins. *Acta Odontologica Scandinavica*.

[31] Takabayashi Y (2010). Characteristics of denture thermoplastic resins for non-metal clasp dentures. *Dental Materials Journal*.

[32] John J, Gangadhar SA, Shah I (2001). Flexural strength of heat-polymerized polymethyl methacrylate denture resin reinforced with glass, aramid, or nylon fibers. *The Journal of Prosthetic Dentistry*.

[33] Radford DR, Sweet SP, Challacombe SJ, Walter JD (1998). Adherence of Candida albicans to denture-base materials with different surface finishes. *Journal of Dentistry*.

[34] Yamauchi M, Yamamoto K, Wakabayashi M, Kawano J (1990). *In vitro* adherence of microorganisms to denture base resin with different surface texture. *Dental Materials Journal*.

[35] Bollen CM, Lambrechts P, Quirynen M (1997). Comparison of surface roughness of oral hard materials to the threshold surface roughness for bacterial plaque retention: a review of the literature. *Dental Materials*.

[36] Berger JC, Driscoll CF, Romberg E, Luo Q, Thompson G (2006). Surface roughness of denture base acrylic resins after processing and after polishing. *Journal of Prosthodontics*.

